# The polypharmacy-xerostomia-nutrition triad: the clinical framework for older adults

**DOI:** 10.3389/fdmed.2026.1900412

**Published:** 2026-07-28

**Authors:** Adam Lowenstein, Mabi L. Singh, Athena Papas

**Affiliations:** Department of Basic and Clinical Translational Sciences, School of Dental Medicine, Tufts University, Boston, MA, United States

**Keywords:** aging, masticatory function, nutrition, older adults, oral health disparities, polypharmacy, salivary hypofunction, sarcopenia

## Abstract

**Background:**

Polypharmacy, the concurrent use of five or more medications, affects the majority of community-dwelling older adults aged 65 and older in the United States. A clinically underrecognized consequence is medication-induced xerostomia and salivary hypofunction, which critically impair oral function, food selection, and nutritional status.

**Objective:**

This mini-review synthesizes mechanistic and clinical evidence linking polypharmacy-induced xerostomia to nutritional decline in older adults and proposes a clinical framework for integrated dental-medical screening.

**Methods:**

A narrative review of English-language peer-reviewed literature published between 2000 and 2025 was conducted using PubMed/MEDLINE, CINAHL, and Cochrane databases. Studies examining xerogenic medication burden, salivary function, masticatory performance, dietary intake, and nutritional biomarkers in adults aged 60 and older were included.

**Results:**

Xerogenic medications, including diuretics, antihypertensives, antidepressants, antihistamines, and anticholinergics, are highly prevalent in older adults and exert dose-dependent suppression of salivary flow via anticholinergic, aquaporin-disrupting, and sympatholytic mechanisms. Salivary hypofunction impairs bolus formation, swallowing efficiency, and gustatory function, driving avoidance of fibrous and nutrient-dense foods. This dietary shift is associated with protein-energy malnutrition, sarcopenia, and micronutrient deficiencies. Socioeconomic and structural disparities amplify nutritional risk among low-income and minority older adults.

**Conclusion:**

The polypharmacy-xerostomia-nutrition triad represents a tractable but systematically under-addressed pathway to preventable malnutrition and functional decline. Interprofessional screening, patient-centered salivary management strategies, and expanded dental benefit coverage are urgently needed.

## 1 Introduction

The global population of adults aged 65 and older is projected to reach 1.6 billion by 2050, accompanied by an increasing burden of multimorbidity requiring complex pharmacological management (1). In the United States, polypharmacy, defined as concurrent use of five or more medications, affects an estimated 36%–42% of community-dwelling older adults, rising to over 65% among those with multiple chronic conditions (2, 3). While the risks of polypharmacy are well-documented across several domains, including falls, drug-drug interactions, and cognitive impairment, one adverse effect remains systematically overlooked in both clinical practice and health policy: the oral consequences of xerogenic medication burden and their downstream effects on nutritional status.

Xerostomia, the subjective sensation of oral dryness, and salivary hypofunction, the objective reduction in salivary flow rate, affect an estimated 25%–35% of community-dwelling older adults and up to 60% of those in long-term care (4, 5). Medications are the leading etiologic factor, implicated in more than 400 commonly prescribed drug classes (6). Despite the centrality of saliva to food intake, digestion, oral comfort, and mucosal protection, xerostomia is routinely dismissed as a mere nuisance rather than recognized as a gateway to nutritional compromise.

This mini-review addresses this gap by presenting a mechanistic and clinical synthesis of the triad linking polypharmacy, xerostomia, and nutritional decline in older adults. We draw on evidence from salivary biology, nutritional epidemiology, and geriatric medicine to construct a pathophysiological framework and to propose targeted interprofessional and policy interventions. This work builds on and extends prior analyses of oral health disparities in aging Americans (7), with emphasis on the nutritional sequelae that disproportionately burden underserved populations.

## 2 The prevalence and pharmacology of xerogenic polypharmacy

### 2.1 Medication burden in aging populations

Polypharmacy has become a near-normative state for older adults managing hypertension, type 2 diabetes, heart failure, depression, chronic pain, and musculoskeletal disorders (8). The American Geriatrics Society Beers Criteria identifies multiple medication classes as potentially inappropriate in older adults, in part due to their anticholinergic and desiccating effects (9). National survey data consistently demonstrate that older adults with polypharmacy have a significantly higher prevalence of xerostomia compared with age-matched peers on fewer medications, independent of salivary gland disease (10).

### 2.2 Mechanisms of xerogenic drug action

Medications induce xerostomia through several converging mechanisms. Anticholinergic agents, the most extensively studied class, block muscarinic M3 receptors on salivary acinar cells, suppressing parasympathetically mediated salivary secretion (11). Diuretics reduce circulating fluid volume and alter electrolyte homeostasis, impairing the aquaporin-mediated water transport essential to salivary gland function (12). Alpha-adrenergic antagonists and certain antidepressants, including tricyclic antidepressants (TCAs) and selective serotonin reuptake inhibitors (SSRIs), modulate sympathetic innervation of serous acinar cells, reducing protein-rich parotid output (13). These mechanisms are dose-dependent and synergistic: a patient receiving a diuretic, an antidepressant, and an antihistamine concurrently faces a substantially compounded xerogenic burden.

Table 1 summarizes the most prevalent xerogenic drug classes in older adult populations, their proposed mechanisms, and the estimated prevalence of use among adults aged 65 and older.

**Table 1:** Commonly prescribed xerogenic drug classes in adults aged ≥65 years. Prevalence estimates derived from national survey data (2, 10, 14).

Drug class	Common agents	Primary mechanism	Prevalence in adults ≥65 (%)
Diuretics	Furosemide, HCTZ	Volume depletion; aquaporin disruption	∼40%
Antihypertensives (ARBs/ACEi)	Lisinopril, Losartan	Reduced secretory drive	∼55%
Antidepressants (TCAs/SSRIs)	Amitriptyline, Sertraline	Anticholinergic; serotonergic	∼20%
Antihistamines	Diphenhydramine, Loratadine	H1 blockade (anticholinergic)	∼15%
Bladder anticholinergics	Oxybutynin, Tolterodine	M3 receptor blockade	∼10%
Antipsychotics	Quetiapine, Haloperidol	Multi-receptor antagonism	∼8%
Opioid analgesics	Oxycodone, Hydrocodone	Reduced parasympathetic tone	∼18%

## 3 Salivary hypofunction, oral processing, and dietary avoidance

### 3.1 The functional role of saliva in nutritional intake

Saliva is not a passive lubricant but an active participant in the nutritional process, performing at least five functions critical to adequate dietary intake: (i) bolus formation and lubrication enabling safe deglutition; (ii) enzymatic pre-digestion of starches via salivary amylase; (iii) solubilization of tastants to enable gustation; (iv) antimicrobial protection of the oral mucosa and dentition; and (v) buffering of oral pH to protect against cariogenic and erosive insults (15, 16). When unstimulated whole saliva flow rate falls below 0.1 mL/min, the clinical threshold for salivary hypofunction, each of these functions is compromised in proportion to the severity of glandular suppression (17).

### 3.2 Masticatory consequences and the texture-avoidance cycle

Reduced salivary lubrication increases friction between food particles and mucosal surfaces and diminishes the cohesiveness of the food bolus required for safe swallowing (18). Patients compensate behaviorally by avoiding foods requiring extensive mastication: raw vegetables, fibrous meats, dense whole grains, and fresh fruits, precisely the foods underpinning micronutrient-rich dietary patterns (19). The result is a texture-avoidance cycle that begins with a dry mouth and leads to food avoidance, dietary monotony, nutritional compromise, and accelerated functional decline.

This phenomenon is well-documented in the denture literature, where edentulous and partially dentate older adults similarly restrict dietary variety due to impaired masticatory efficiency (20). When xerostomia and tooth loss co-occur, as they frequently do in older adults, the nutritional impact is amplified. Longitudinal data from the Normative Aging Study and the Veterans Affairs Dental Longitudinal Study suggest that masticatory limitation, regardless of etiology, is independently associated with lower intakes of dietary fiber, vitamins C and B12, folate, and iron (21).

### 3.3 Gustatory impairment and appetite dysregulation

Tastants such as sugars, salts, acids, and glutamates must be solubilized to bind taste receptor cells in lingual taste buds (22). Reduced salivary flow impairs this solubilization step, attenuating taste signal transduction. Select medications, including ACE inhibitors, certain antihypertensives, and chemotherapeutic agents, additionally alter taste receptor physiology directly (23). The net effect is diminished food palatability, reduced appetite, and a behavioral shift toward higher-calorie, intensely flavored processed foods to compensate for attenuated sensory experience (24).

## 4 Nutritional consequences in older adults

### 4.1 Protein-energy malnutrition and sarcopenia

Malnutrition is present in approximately 15% of community-dwelling older adults and 35%–65% of those in institutional settings (25). Oral functional limitation, including xerostomia-driven dietary avoidance, is an underweighted contributor to this burden. Inadequate protein intake, particularly below 1.0 g/kg body weight per day, accelerates skeletal muscle loss (sarcopenia), impairing physical function, increasing fall risk, and contributing to frailty (26). The soft, processed dietary patterns that xerostomic older adults gravitate toward tend to be calorie-sufficient but protein- and micronutrient-poor, a constellation sometimes characterized in the geriatric literature as obesogenic malnutrition (27).

### 4.2 Micronutrient deficiencies and systemic sequelae

Avoidance of fibrous and textured foods reduces intake of multiple micronutrients with direct relevance to aging health. Vitamin C deficiency, associated with restricted fruit and vegetable consumption, impairs collagen synthesis and immune function and is associated with accelerated periodontal breakdown (28). Deficiencies in vitamin D and calcium, arising in part from avoidance of dairy products (often perceived as mucus-forming in xerostomic patients), accelerate osteoporosis and fracture risk (29). Folate and vitamin B12 deficiencies, arising from reduced intake of leafy greens and lean meats, contribute to cognitive decline and megaloblastic anemia (30). These effects compound over time, and their dietary origins, rooted in part in oral functional limitation, are rarely recognized in medical nutrition therapy.

### 4.3 Evidence from longitudinal and cross-sectional studies

Prospective data directly linking xerostomia to nutritional outcomes remain limited but growing. A longitudinal study by Ikebe et al. among Japanese community-dwelling older adults found that decreased salivary flow at baseline predicted significantly lower dietary variety scores at five-year follow-up, independent of tooth number and denture status (31). Cross-sectional analyses from the National Health and Nutrition Examination Survey (NHANES III) have demonstrated significant associations between dry mouth symptoms and lower serum levels of vitamins C and B12 and reduced serum albumin, suggestive of protein deficiency (32). Foundational longitudinal cohort data from the Papas laboratory at the Tufts University School of Dental Medicine (TUSDM) provide a unique U.S.-based resource for examining oral-nutritional associations in diverse, aging urban populations (7, 33).

## 5 Health disparities and inequitable burden

The intersection of polypharmacy, xerostomia, and nutritional vulnerability is not distributed equitably. Socioeconomic disadvantage, racial and ethnic minority status, rural residence, and limited English proficiency are independently associated with higher medication burden, lower dental care utilization, and greater food insecurity among older adults (7, 34). Low-income older adults are simultaneously more likely to carry high xerogenic medication loads, reflecting elevated rates of hypertension, diabetes, and depression, and less likely to have access to dental care or salivary management interventions.

The historical absence of a comprehensive dental benefit in traditional Medicare left older adults without coverage for the preventive and restorative care that could mitigate oral functional decline. The partial expansion of Medicare dental benefits in 2024 represents a meaningful but insufficient step toward equity. Food insecurity further compounds nutritional risk: older adults with limited food access cannot compensate for xerostomia-related dietary restrictions by diversifying their dietary supply. Research from the Boston Elders Nutrition and Dental Health (BEND) cohort at TUSDM demonstrated that the combination of oral functional limitation and food insecurity was a significantly stronger predictor of nutritional inadequacy than either factor alone (33). These findings underscore the imperative for equity-informed dental public health policy.

## 6 A clinical framework for identification and management

### 6.1 Interprofessional screening

Effective identification of at-risk older adults requires interprofessional collaboration across pharmacy, medicine, and dentistry. Pharmacists conducting medication therapy management reviews should systematically flag high anticholinergic burden using validated instruments such as the Anticholinergic Cognitive Burden (ACB) Scale or Drug Burden Index (DBI) (34). Primary care clinicians performing annual wellness visits should incorporate a brief xerostomia screening question, such as items from the validated Xerostomia Inventory, with referral to dental providers when positive (35). Dental clinicians should perform salivary flow measurement, conduct medication reconciliation, and apply a brief nutritional risk tool such as the Mini Nutritional Assessment (MNA) or DETERMINE Checklist (36).

### 6.2 Salivary management and dietary counseling

Management of medication-induced xerostomia in older adults is primarily palliative but can meaningfully improve function and quality of life. Evidence-supported strategies include: stimulation of residual salivary function with xylitol-containing gums or lozenges in patients with preserved acinar tissue; use of salivary substitutes (carboxymethylcellulose- or mucin-based) for patients with severe hypofunction; topical fluoride application and antimicrobial rinses to reduce caries and periodontal risk; and physician consultation for deprescribing or therapeutic substitution of the most xerogenic agents where clinically feasible (37). Dietary counseling should emphasize texture modification strategies, including steaming, slow cooking, and sauce supplementation, that restore the palatability and intake of nutrient-dense foods without requiring intact salivary function (38).

### 6.3 Policy imperatives

Systemic solutions require policy action. While the 2024 partial Medicare dental benefit expansion is a meaningful first step, parity with medical benefits, encompassing salivary diagnostics, implant-supported prosthetics, and therapeutic xerostomia management, remains a policy priority. Integration of oral health screening into annual Medicare wellness visits, Medicaid managed care contracts, and federally qualified health center (FQHC) service models would extend interprofessional xerostomia-nutrition screening to the populations most vulnerable to its consequences (7, 39).

## 7 Conclusion

Polypharmacy-induced xerostomia is a prevalent, mechanistically well-characterized, and clinically tractable gateway to nutritional deficiency in older adults, yet it remains systematically underaddressed across medicine, dentistry, and nutrition practice. The convergence of xerogenic medication burden, impaired oral function, dietary avoidance, and nutritional compromise constitutes a triad that accelerates functional decline and widens health disparities in aging populations. Bridging this divide requires investment in interprofessional education, validated screening workflows, and equitable dental benefit policy. Longitudinal research, particularly in diverse, socioeconomically heterogeneous aging cohorts that track xerostomia severity alongside dietary patterns and nutritional biomarkers, is urgently needed to quantify this pathway's contribution to preventable decline and evaluate the effectiveness of integrated interventions.
